# Dietary Composition and Effects in Inflammatory Bowel Disease

**DOI:** 10.3390/nu11061398

**Published:** 2019-06-21

**Authors:** Fernando Castro, Heitor S. P. de Souza

**Affiliations:** 1 Department of Internal Medicine, School of Medicine, Federal University of Rio de Janeiro, 21941-913 Rio de Janeiro, Brazil; fernandommcastro@gmail.com; 2 Department of Internal Medicine, D’Or Institute for Research and Education (IDOR), 22281-100 Rio de Janeiro, Brazil

**Keywords:** inflammatory bowel disease, dietary habits, food components, gut microbiota, immune homeostasis, epigenetic changes

## Abstract

Dramatic changes in the environment and human lifestyle have been associated with the rise of various chronic complex diseases, such as inflammatory bowel disease (IBD). A dysbiotic gut microbiota has been proposed as a crucial pathogenic element, contributing to immune imbalances and fostering a proinflammatory milieu, which may be associated with disease relapses or even the initiation of IBD. In addition to representing important regulators of the mucosal immunity and the composition of the gut microbiota, food components have been shown to be potential environmental triggers of epigenetic modifications. In the context of chronic intestinal inflammation, dietary habits and specific food components have been implicated as important modulators of epigenetic mechanisms, including DNA methylation, which may predispose a person to the increased risk of the initiation and evolution of IBD. This review provides novel insights about how dietary factors may interact with the intestinal mucosa and modulate immune homeostasis by shaping the intestinal ecosystem, as well as the potential influence of diet in the etiopathogenesis and management of IBD.

## 1. Introduction

Inflammatory bowel disease (IBD), including Crohn’s disease (CD) and ulcerative colitis (UC), is regarded as a heterogeneous disease of unknown etiology, with an unpredictable clinical course and usually labeled as a chronic disabling disorder [1,2]. Although the mechanisms leading to the development of IBD are rather complex and have not yet been completely elucidated, the current information points to a multifactorial origin. The chronic inflammatory process underlying the pathogenesis of IBD reflects a dysregulated immune response, stemming from a conflicting interaction involving several environmental factors, an altered innate immunity, and a predisposing genetic background [3,4,5,6].

Information derived from epidemiological studies has revealed consistent associations between the increase in the occurrence in IBD and other autoimmune and chronic inflammatory disorders, and the decline in infectious diseases, accompanied by the use of antibiotics, immunizations, and overall improvements in sanitary conditions and the quality of water [7]. IBD has progressively become a global disease, affecting newly industrialized nations in Asia, South America, and the Middle East [8,9,10]. Although the prevalence of IBD in newly industrialized countries is still lower than in developed countries, the incidence rate is increasing rapidly [11]. Among all the environmental changes usually associated with IBD, dietary habits are one element, both from the patients’ perspective, including personal observations and common practical concerns, and due to scientific evidence implicating diet as a triggering factor in disease relapses [12]. In this article, we discuss the effects of diet on IBD, especially considering the basic physiological processes that underlie these associations.

## 2. Diet and Microbiota

Although the influence of dietary habits on the intestinal microbiota has long been speculated, only after the introduction of new technology, including next-generation DNA sequencing and metabolic profiling, was reliable information able to be obtained [13]. For example, the microbiota composition is currently thought of as a dynamic process that changes with age and fluctuates in consonance with environmental exposures, including diet, among other factors [14].

The results of studies analyzing the microbial communities of the gut obtained from mammalian species have endorsed the assumption that diet can influence microbial diversity. Such diversity has been shown to increase from carnivores to omnivores and then to herbivores [15]. Regarding humans, the intestinal microbiota reflects predominant omnivorous habits, but substantial inconsistencies have been reported. For example, opposing patterns of the intestinal microbiota composition were reported when comparing samples from African children living in rural communities with children from a European urban center, a phenomenon that has been attributed to dietary differences [16]. In this case, in terms of health issues, individuals from the European urban center are expected to be at increased risk for the development of several immune-mediated disorders. Such diseases, likely including IBD, are probably associated with the microbiome structure, often resulting from dietary incompatibilities between the current environments and those in which humans evolved. 

The Western style of diet, defined by high caloric content due to large amounts of fat and carbohydrates, has been associated with a markedly reduced microbiome diversity [17]. In addition to the plethora of fat, Western diets usually have high concentrations of dietary omega-6 fatty acids, from vegetable oils, resulting in a high omega-6 to omega-3 ratio. Whereas omega-3 fatty acids, such as a-linolenic acid from vegetables and eicosapentaenoic acid and docosahexaenoic acid from fish, have anti-inflammatory properties, omega-6 fatty acids, particularly arachidonic acid, are considered pro-inflammatory. This may explain why the Western diet has been regarded as a key factor for producing intestinal inflammation [18]. Other relevant studies focusing on diet potentially affecting the intestinal microbiota are summarized in Table 1.

Previous studies have analyzed the gut microbiota of infants and children within one population, but few have attempted to compare the microbial composition considering distinct socio-economic, geographic, and cultural aspects [16]. To analyze the potential effect of environmental factors on defining how and from where a gut microbiota may be acquired, investigators characterized bacterial species and gene contents of fecal samples from a cohort of healthy Amerindians from Venezuela, rural Malawian communities, and metropolitan USA inhabitants. The investigators found that bacterial diversity increases with age, and the degree of similarity among members of a family is consistent in all three populations. The fecal microbiota of USA adults was the least diverse compared to the others [19]. Therefore, it appears that differences in social structures and cultural traditions known to impact food and many other environmental factors may also condition the magnitude of the circulation of microbes among members of a family or community.

## 3. Diet and Immune Response

Western lifestyle factors have been associated with the progressive increase in various metabolic, autoimmune, and chronic inflammatory disorders, including IBD [32,33,34]. In addition to industrialization, urbanization, the improvement of sanitary conditions, antibiotic usage, and other factors, abundant evidence supports the involvement of dietary habits in shaping the gut microbiota and influencing the interaction with the immune system [35,36,37]. 

Dietary fiber has been shown to interfere in host immunity via multiple pathways. Fibers and starches present in fruits and vegetables represent a substrate for the production of butyrate and other short chain fatty acids (SCFAs) by intestinal microbiota. Butyrate, for example, is well-recognized as a critical element in epithelial homeostasis, but also plays an important role in downregulating the immune response by inhibiting the transcription of inflammatory cytokines and promoting the differentiation of lamina propria Tregs [5,38,39]. Low dietary fiber may precipitate the catabolism of the mucous layer, leading to increased permeability and compromising the epithelial barrier against potential pathogenic luminal bacteria [40].

Investigations based on experimental models have provided results regarding the effects of specific diets on the immune response. For instance, a high-fat (HF) and high-sugar diet generated a proinflammatory intestinal environment, associated with an overgrowth of Proteobacteria, reduction in butyrate production, and expression of the butyrate GPR43 receptor, also underexpressed in CD patients [41]. Results from another experimental model showed that HF diets led to the upregulation of tumor necrosis factor-α (TNF-α) and interferon-γ expression, and to the decrease in the densities of colonic Tregs [42]. A high intake of fat has been shown to have several effects on the mucosal immune system, revealing the complex interaction between the lamina propria immune cells and the intestinal microbiota. For example, the ingestion of high-fat meals increases the expression of interlukein-1β (IL-1β), TNF-α, IL-6, and necrosis factor-κB (NF-κB) in the colon [43,44]. Likewise, mice fed with cholesterol-enriched meals displayed acute inflammasome-dependent intestinal inflammation, increasing the production of IL-1β, and driving the accumulation of CD11b+ and CD11c+ cells in the lamina propria [45]. 

The increasing knowledge about the influence of the gut microbiome on the mucosal and systemic immune response has contributed to the understanding of the mechanisms underlying the pathogenesis of IBD and other chronic inflammatory conditions. Dysbiosis has been confirmed in samples from patients with CD, displaying an increase in Enterobacteriales such as *Escherichia coli*, and a decrease in Clostridiales such as *Faecalibacterium prausnitzii* [46,47,48]. In a study involving UC, the results supported a role for reduced microbial diversity, although not as prominent as the dysbiosis seen in CD [49]. A large cohort study analyzing fecal samples from four different European countries revealed that CD and UC are distinct in terms of the microbiome [50]. Notably, some specific intestinal microbes can shape the immune response. For example, *Listeria monocytogenes*, *Clostridium difficile*, and *Toxoplasma gondii* have been shown to elicit innate lymphoid cells-1 (ILC1) and Th1 cells, which orchestrate the secretion of cytokines, such as interferon-gamma and TNF-α, critically important in the immunity against those intestinal pathogens [51]. Clostridia strains have been shown to promote the accumulation of Foxp3 Treg cells in the gut [52], which in turn down-regulate inflammatory responses, including those in experimental colitis [53]. Some microorganisms induce the activation of subsets of immune cells responsible for promoting intestinal inflammation, such as Th17 cells [54]. For instance, segmented filamentous bacteria can activate Th17 cells, whereas a decrease in Th17 cell-inducing bacteria can attenuate the severity of colitis in mice [55]. In previous studies, adherent invasive *E. coli* (AIEC) and *Bifidobacteria adolescentis* [56,57], in addition to *E. coli O157* and *Citrobacter rodentium* [54], have been shown to induce populations of Th17 cells.

Dietary habits can affect human health either directly or via changes in the gut microbiota. The gut microbiota is a highly complex and dynamic system that can be shaped by environmental factors, including the diet [58]. Regarding the dietary factors directly or indirectly associated with the immune response and intestinal homeostasis, diets with high concentrations of fat and sugar have been associated with intestinal colonization with potential IBD-related pathobionts. For instance, adherent invasive *E. coli* (AIEC) strains, induced by high-fat high-sugar diets, may become pathogenic when the ligand CEACAM 6 is presented by the intestinal epithelium. Under such circumstances, AIEC may form biofilms, adhere to, and translocate via M cells and the associated epithelium [59]. These findings reinforce the idea that changes in the local environment exert a selective pressure on several species, which in turn shape the microbiota according to the most adaptable microorganisms to the intestinal milieu [60]. A selection of dietary factors that may affect the host immunity is listed in Table 2.

## 4. Diet and IBD 

Advances resulting from clinical and experimental investigations have demonstrated that the microbiome might play a critical role in the pathogenesis of IBD [65,66]. In parallel, results from other studies have supported a link between dietary patterns and the structure of the intestinal microbiome in healthy individuals and in patients with gastrointestinal disorders, including IBD [17,27,67]. Given the potential effects that diet can have on the composition and function of the intestinal microbiome, diet may also be involved in the pathogenesis of IBD, either directly or indirectly. As a consequence, due to the associations with the microbiome and IBD, specific dietary interventions are promising novel therapies. 

Data have confirmed that food plays a crucial role in the gut microenvironment, and modifications of dietary habits may have an important impact on the microbial composition and function, but also on the gut barrier and immunity [5]. Previous studies demonstrated that modifications in a single food group might produce various outcomes. For instance, changing the dietary composition from a predominant plant- to animal-based diet results in a change in the intestinal microbial environment and functionality, determining alterations in bile acids and sulfide metabolism, which have been implicated in IBD pathogenesis [27,68]. In an experimental study, investigators demonstrated that a low-fiber diet induces the expansion and activity of colonic mucous-degrading microorganisms, potentially favoring the development of severe colitis by enteric pathogens [40]. In another experimental study, mice fed a Western-style diet (rich in saturated fats and simple carbohydrates, but depleted in dietary fiber), developed an alteration in the colonic microbiota, resulting in increased permeability and a reduced inner mucus layer. Both defects could be prevented by transplanting microbiota from chow-fed mice, whereas the administration of *Bifidobacterium longum* was sufficient to restore the mucus layer [69]. These findings suggest that specific bacteria are crucial for normal mucus and barrier function and, in humans, this information could help us to understand important mechanisms underlying IBD pathogenesis. 

Unveiling the association between diet and the development of IBD would ideally require knowledge on dietary details before the onset of the disease. In this regard, large longitudinal studies have been performed that have started to provide information to support specific dietary patterns as risk factors for IBD [17]. For instance, the metabolic activity of the microbiota may present important abnormalities in IBD. A reduced production of SCFAs, which are promoted by the presence of dietary fiber, has been shown in UC and CD. Particularly in patients with UC, a deficient production of SCFAs has been shown, and could not be corrected by increasing the intake of wheat bran-associated fiber and high amylose-associated-resistant starch [5,70].

Among the dietary factors potentially implicated in the development of IBD, dietary fiber has received special attention due to evidence showing associated protective mechanisms, including the conversion to SCFAs [71,72], interference in the structure of the gut microbiota [16], and maintenance of the barrier function [73]. However, a large prospective cohort study evaluating the impact of the intake of dietary fiber from several sources failed to identify any protective effect against the development of IBD [74].

Regarding the presumably harmful Western diet, in addition to its general composition characterized by high animal or dairy fat and animal protein, it is important to draw attention to the ubiquitous presence of emulsifiers, thickeners, and artificial sweeteners, many of which are associated with abnormal intestinal permeability, dysbiosis, and intestinal inflammation in animal models [75,76]. As an integral part of this type of diet, the common use of processed foods, usually rich in phosphate [77], but poor in micronutrients such zinc and other nutrients including omega-3 fatty acids, and vitamins D and E [78,79,80], may also be involved in the development or the predisposition to chronic intestinal inflammation.

## 5. Environmental Factors and IBD

The rise of IBD in the developing world has usually been attributed to the industrialization and urbanization of societies [11,81]. The observation of the coincident increase in immune-mediated and inflammatory diseases in the more developed and industrialized nations has long been associated with the hygiene hypothesis [82]. This hypothesis appears to be relatively reductionist, as it does not explain the dynamics of IBD evolution in long-modernized Asian countries, for example. Whereas improvements in sanitation and hygiene practices are usually related to the urban compared to the rural environment, several other concomitant factors affecting the rise of immune-mediated disorders need to be pondered. For example, areas with a higher prevalence of IBD also have fewer infections and are characterized by the widespread use of vaccines and antibiotics, in addition to the availability of clean food and water [83,84,85]. Other considerations associated with modern urban life in developed and developing nations include, for instance, population density, lifestyle modifications, level of education, and exposure to pollutants [86]. A positive association between ambient air pollution and IBD has been previously suggested [81], and the residential exposures to nitrogen dioxide and sulfur dioxide were shown to increase the risk of early-onset IBD [87]. 

The increased risk for developing IBD has also been associated with the exposure to different medications. Among them, the use of antibiotics has been shown to increase the risk of IBD, especially CD [88,89]. In a nested case-control analysis of the population-based database of prescription drugs, investigators found that patients diagnosed with IBD in childhood were more likely to have used antibiotics during the first year of life [90]. This positive association was further corroborated, for example, in a prospective study in which the use of antibiotics was strongly associated with the development of CD in childhood [91]. Although causality cannot be firmly established to date, exposure to antibiotics in childhood has been proposed to affect the normal development of tolerance to enteric bacteria, increasing the risk of IBD [92].

Other medications have been linked to IBD. For example, oral contraceptives have been shown to have a positive association with the development of IBD, in particular with CD [93]. In a case-control study, investigators found a positive association between nonsteroidal anti-inflammatory drugs (NSAIDs) and IBD [94]. In a cohort study, investigators found that the regular use of NSAIDs may increase CD activity [95]. However, a systematic review did not find a reliable association between the use of NSAIDs and risk of CD and UC exacerbation [96]. NSAIDs are capable of disrupting the intestinal epithelial barrier, and this may predispose the individual to the invasion of pathogens and may affect the intestinal homeostasis. The conflicting results regarding the association between NSAIDs and IBD may reflect different methodologies but also the participation of other uncontrolled environmental factors generating unanticipated and confounding effects.

Smoking is a well-established modifying factor in IBD, with opposing effects in CD and UC [97], but the mechanism by which it regulates intestinal inflammation is still unclear. The composition of cigarettes is heterogeneous and complex with several chemical elements that may affect different targets in the intestinal cells and modulate the microbiota [98,99]. Other evidence suggests that chemicals released from cigarette smoking can interfere with functions related to the immune response, such as the production of cytokines [100], and significantly impact the microbiota [101].

The functionality of vitamin D, an important regulator of mucosal immunity, is critically related to the exposure to sun with natural UV light [102]. Therefore, low sunlight exposure has been proposed to be a risk factor for the development of IBD, especially CD [103,104]. This is in line with the incidence and prevalence rates of IBD being greater in the Northern Hemisphere, where UV light is markedly lower [105]. However, the proposed association between IBD, particularly CD, and the northern latitudes should be interpreted not only in terms of geography, but in a wider context, where several other variables interact in a complex and dynamic concert, with largely unpredictable outcomes. 

## 6. Epidemiological Associations between Diet and IBD

Although a consistent correlation between environmental factors and the pathogenesis of several disorders is usually difficult to prove due to the intervening effect of potentially confounding variables and the uncertain dynamics of exposures, associations between IBD and diet have been reported. The realization that the increase in the worldwide incidence of IBD and other immune-mediated diseases began in more developed and industrialized countries, particularly in North America and Northern Europe, has led investigators to hypothesize the global spread of a “Westernized” lifestyle [106,107,108].

The observation that the risk of developing IBD increases when people move from a low-risk to high-risk area appears to further support the role of environmental factors in the detriment of genetic predisposition, and reinforces the utility of studies examining immigration. For example, distinct investigations showed that immigrants to Israel had a higher prevalence of IBD than populations born in Israel [107,109]. Another population-based study demonstrated that in Sweden, a high incidence region, second-generation but not first-generation immigrants developed IBD at rates comparable to the native Swedish population. These results appear to underscore the importance of early-life exposures in determining disease initiation [110]. 

Epidemiological studies investigating the South Asian population indicate that the incidence and prevalence of adult IBD are significantly lower than in North America or the U.K. [111,112,113,114]. However, higher incidences of IBD in patients from South Asia have been reported in adult individuals who subsequently migrated to the U.K. [115], and in children migrating to British Columbia, Canada, at rates higher than in other ethnicities, including children of Western European descent [116]. In another similar study analyzing a migrant group to investigate the role of environmental factors in the development of IBD, Middle Eastern migrants to Australia had a significantly higher risk compared with the Caucasian population. The results, including the analysis of additional exposures such as smoking, antibiotic usage, and hygiene markers, suggested that migrants might be more sensitive to environmental challenges influencing the gut microbiome [117]. This thought is further supported by the results of a population-based study in the Asia-Pacific area in which the investigators suggested that early childhood factors and markers of altered intestinal microbiota may modulate the risk of IBD later in life [118].

Another study analyzing an Asian population combined a series of Chinese patients with IBD with the sequences from RISK and PRISM cohorts of IBD patients from the United States for a meta-analysis comparing gut microbiome profiles across ethnic groups. The investigators found that the gut dysbiosis observed in IBD is similar among Chinese and Western populations [119]. This may lead to the question of whether the gut microbiota provides universal biomarkers in IBD regardless of ethnicity. Results from another study suggest the existence of diverse gut microbial taxa with differential patterns of abundance common to various immune-mediated inflammatory diseases (IMIDs), including IBD [120]. These findings appear to corroborate the possible use of microbial taxa patterns as biomarkers for the detection and diagnosis of IBD, but also support the idea that the gut microbiota may constitute a common component of the etiology of IMIDs. 

Several observational studies have attempted to characterize dietary patterns that could contribute to a higher risk of IBD development. These studies pointed to an increased risk of IBD among people consuming larger proportions of meat and fats, and a lower risk among people with diets rich in fiber, fruits, and vegetables [121,122]. However, as in other complex diseases, it may be difficult to interpret the role of individual risk factors, and an analysis of dietary patterns should consider exposures to groups of foods [5], distinct food components, and cultural habits, including specificities in food preparation. 

## 7. Diet and Epigenetic Modifications in IBD 

The epigenome, an intersection between the environment and the genome, has been implicated in the regulation of gene expression and cellular functions, and plays an essential role in the delineation of phenotypes and their preservation [21]. Novel information has contributed to an improved understanding of the role of epigenetic modifications in defining the molecular basis of IBD [123,124]. Regarding IBD, the first epigenetic modifications associated with disease pathogenesis were based on DNA methylation studies [125]. 

Currently, among the environmental factors, dietary factors were found to be powerful stimuli, being associated with peculiar patterns of gene expression and epigenetic signatures [126]. For instance, the diet supplies substrates for DNA methylation and can regulate the enzymatic activity required in the one-carbon cycle. Consequently, elements such as folate, choline, and water-soluble B vitamins have been implicated in methylation patterns [123,127], which are essential elements for the synthesis and repair of DNA that modulate gene expression [128]. Experimental evidence obtained also supports the idea that dietary nutraceuticals possess potential as epigenetic modulators. For example, data from studies involving DNA methylation and chromatin repair indicate that polyphenols can regulate gene expression [129]. Polyphenols are secondary plant metabolites, common components of vegetables, fruits, green tea, and red wine, and have been recognized for their antioxidant properties. In addition, studies have shown beneficial effects of polyphenols through the modulation of NF-κB expression, and chromatin remodeling via the modulation of histone deacetylases and DNA methyltransferase activities, reversing altered gene expression [130]. In experimental IBD, resveratrol, a natural phenolic compound, was shown to decrease inflammatory cytokines and profibrotic factors, supporting a potential therapeutic benefit [131].

Curcumin, another natural product commonly used in cooking, has been investigated in experimental models of IBD, demonstrating beneficial effects mediated by the suppression of proinflammatory mediators [132]. In a randomized, multicenter, double-blind, placebo-controlled trial, curcumin has shown beneficial results in maintenance therapy for patients with UC [133]. Although the exact mechanisms by which curcumin may exert a wide range of biological actions have not been established, effects have been attributed to the regulation of histone acetylation/deacetylation and the expression of various microRNAs [134]. 

Several nutrients have been shown to modulate immune responses, potentially counteracting inflammatory processes [135], in actions also mediated through epigenetic regulation [123,136]. Processed foods, deficient in micronutrients including selenium and folate, have been implicated in the progression of diseases, such as colorectal cancer and possibly IBD [21,137,138,139]. In an experimental model, a selenium-deficient diet was shown to result in markedly hypomethylated colon DNA [140]. Selenium supplementation was able to prevent tissue damage by modulating the expression of key genes responsible for inflammation in experimental IBD [141]. Although low levels of selenium have been detected in the serum of patients with IBD [142], studies in human IBD involving potential effects of selenium supplementation are still limited [21]. 

To investigate the mechanisms underlying the potential beneficial effects of the Mediterranean diet, an intervention with two different diets, one rich in nuts and the other one in extra-virgin olive oil, was conducted. The evaluation of the methylation status of peripheral white blood cells genes showed that both diets influenced methylation, characteristically of genes such as *CPT1B* and *GNAS* involved in intermediate metabolism, inflammation, and the signal transduction process. Therefore, the investigators concluded that the expected beneficial effects of the Mediterranean diet, supplemented with nuts or extra-virgin olive oil, could be mediated, at least in part, through epigenetic modifications [16,143,144].

**Figure 1:**
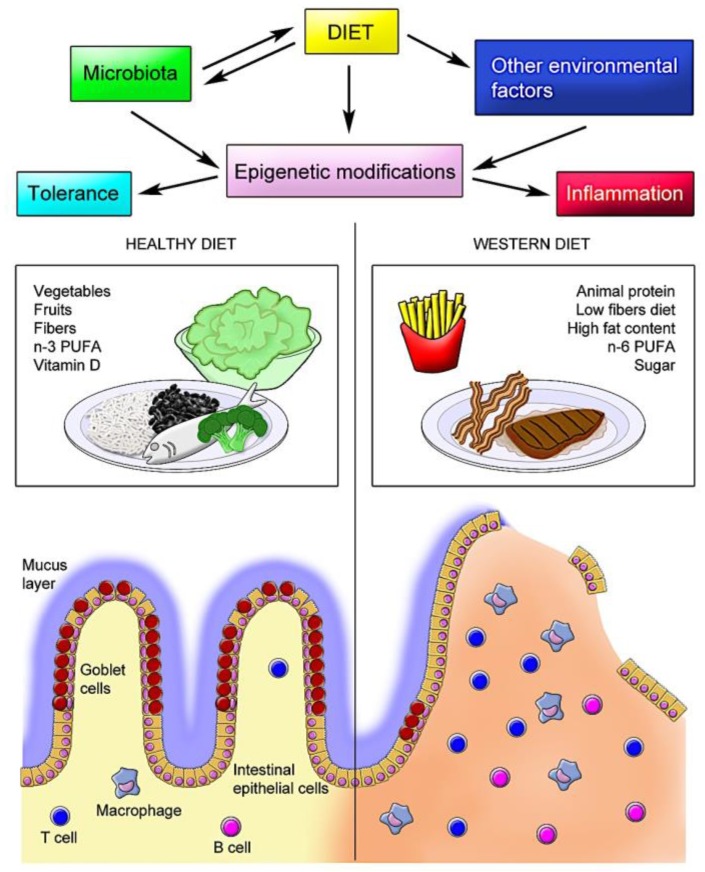
Schematic model of diet–host interactions in the intestine. The interaction between dietary elements and the intestinal mucosa is highly complex and, in normal conditions, results in a tolerogenic response. However, in genetically predisposed individuals, the interplay of specific dietary constituents with the resident microbiota and other environmental factors induces epigenetic modifications that affect the immune response, further compromising the epithelial barrier and defense mechanisms, leading to chronic inflammation, as observed in inflammatory bowel disease (IBD).

In addition to the direct effects of dietary constituents on epigenetic modifications in human intestinal tissues, the resident microbiota has been shown to alter host histone acetylation and methylation. In this regard, particularly short-chain fatty acids such as acetate, butyrate, and propionate, mostly produced by the microbial fermentation of fiber, appear to play a critical role in the epigenetic control of the inflammatory response. In a diet poor in fiber, the inhibition of SCFA production was correlated with disturbed chromatin effects, which were restored following supplementation with SCFA [145]. In human IBD, SCFA-producing bacteria (*Roseburia*) and butyrate-producing bacteria (*Faecalibacterium*) are reduced [146]. However, the application of butyrate-producing bacteria as nutraceuticals in humans has been questioned and needs further investigation [123,147]. Figure 1 summarizes the potential interactions between diet and the microbiota and other environmental factors, resulting in epigenetic modifications, exemplifying the effects of different diets with their respective major characteristics.

## 8. Clinical Effects of Dietary Factors 

Clinical experience shows that patients usually associate their symptoms or disease relapses with the ingestion of certain foods and, as a result, they often change their diet empirically, with variable and, at most, temporary effects. In practice, decisions on dietary modifications usually do not follow professional nutritional advice and may have detrimental consequences [17,148,149,150]. In a prospective study investigating the dietary influence in the course of UC, researchers observed that a large ingestion of meat, more so of red and processed meat, in addition to ethanol, protein, sulfur, and sulfate, enhanced the chances of triggering a flare [151,152]. In the case of patients with CD, a diet rich in total fat, saturated fat, monounsaturated fatty acids, and a higher ratio of omega-6:omega-3 polyunsaturated fatty acids (PUFAs) was related to disease relapses [153,154]. Of note, the oral administration of iron sulfate or heme in dietary iron, which is present in meat, was shown to increase the severity of chemically-induced colitis in rodents [155,156]. However, although a substantial number of iron-deficient patients tolerate oral iron poorly, only a small percentage of patients with IBD have disease relapses attributed to oral iron [157]. Anemia is a common complication of IBD, mostly multifactorial, but frequently related to iron deficiency due to loss from bleeding and decreased iron absorption or ingestion [158]. Although many physicians have concerns regarding the management of iron deficiency in IBD, current data suggest that oral iron therapy should be preferred in mild cases or during clinical remission, unless patients are intolerant or have an inadequate response [159]. The aggravation of experimental colitis following dietary iron has been associated with a marked reduction in the fecal abundance of Firmicutes and Bacteroidetes, and an increase in Proteobacteria [160]. Hence, poor outcomes following oral iron replacement in patients with IBD could be related to exacerbation of the intestinal dysbiosis. 

## 9. Dietary Interventions in IBD

Simple dietary interventions usually improve gastrointestinal symptoms in patients with IBD, including a reduction in the ingestion of dairy products and also fermentable oligosaccharides, disaccharides, monosaccharides, and polyols (FODMAPS) [161]. More recent data have demonstrated the possible roles of specific dietary therapy in IBD. For example, dietary therapies, such as exclusive enteral nutrition (EEN), have been successfully used to induce remission in early or new-onset CD [162]. Theoretically, EEN involves the isolated use of medical formulas in a liquid form without exposure to other foods, usually for six to eight weeks [5]. This strategy is apparently more feasible in the pediatric population, in contrast to adults. Investigations have demonstrated that EEN may induce remission in more than 60% of children with CD followed by a significant reduction in inflammatory markers, including the erythrocyte sedimentation rate (ESR), C-reactive protein (CRP), and fecal calprotectin [5,163,164,165]. Grover et al. conducted a prospective open label study to assess mucosal healing before and after eight weeks of EEN, and concluded that the therapy is effective for inducing early clinical, biochemical, mucosal, and transmural remission, and was associated with improved outcomes at one year [166]. 

Given practical concerns and clinical limitations, other research groups have employed a different strategy based on the combination of partial enteral nutrition (PEN) with a specific exclusion diet granting access to whole foods. The diet was evaluated in a retrospective cohort of children and young adults with mild-to-moderate CD, and remission was achieved in 70% of patients. Clinical improvement was followed by the reduction in ESR and CRP and was associated with mucosal healing [167]. Afterward, this diet—low in animal fat, rich in complex carbohydrates, and with a moderate exposure to soluble fiber—was evaluated for the induction of remission in children and adults failing biologics. More than 60% of the patients achieved clinical remission, with a marked decrease in inflammatory markers [168]. 

Other important theoretical dietary elements, fiber, and prebiotics supplements have been evaluated as interventions in CD. However, a systematic review of randomized controlled trials found no evidence for the use of fiber or prebiotics to induce or maintain remission in CD [169]. No evidence for the restriction of fiber in the absence of obstructive symptoms or stricturing disease has been established for CD. In another study, a semi-vegetarian diet with considerable amounts of dietary fiber was shown to induce a high remission rate with no negative effects [170]. The same group investigated the effect of infliximab combined with a plant-based diet as first-line therapy for patients with CD naïve to biologics and found that the combined therapy can induce remission in most cases [171]. 

Although the most consistent studies regarding dietary interventions in IBD usually refer to CD, previous systematic evaluations performed by independent groups have supported the potential beneficial effects of diet in both CD and UC according to patients’ perceptions [149,172]. In an observational prospective cohort study, patients with UC in clinical remission were followed for one year to investigate the effect of diet on disease relapse. The investigators identified an increased risk of UC relapses associated with the consumption of meat (particularly red and processed meat), eggs, and alcoholic beverages, which have been attributed to the high dietary content of sulfur [151]. In addition to red meat and eggs, milk and dairy products contain high concentrations of cysteine, which can be used by sulfate-reducing bacteria (SRB) to produce hydrogen sulfide (H_2_S) [173,174]. H_2_S, in turn, has been shown to induce proinflammatory effects in the colon and intestinal epithelial cells [175,176,177]. Some studies have demonstrated the increased presence of SRB and H_2_S production in the colon of patients with UC [178,179,180]. However, despite all evidence suggesting a link between sulfur or sulfate-containing foods and UC, current data do not allow a causal association. While more controlled studies regarding SRB and H_2_S in UC are awaited, patients should be aware of the potential detrimental effects of high-sulfur-containing dietary products.

The nutritional theories of IBD pathogenesis involving carbohydrates, for example, have been based on the increasing evidence of the beneficial effects of the Specific Carbohydrate Diet in the treatment of both CD and UC, resulting in improvement in clinical parameters and inflammatory biomarkers [181,182,183]. The hypothesis that carbohydrate variation increases the predisposition to IBD has been supported by the evidence of resultant immune dysfunction, mucosal barrier defects, and dysbiosis, which are represented by the Westernized dietary features [1,184,185]. Carbohydrate monotony could be beneficial in IBD [186]. This has been corroborated by a successful report using the Paleo diet for a small cohort of patients with IBD [187]. Paleo is also an exclusion diet, supposedly representing habits of the Paleolithic era, consisting of a non-cereal, plant-based diet, including non-domesticated meats, and a low intake of carbohydrates, which are taken from a monotonous source. The rational for the use of carbohydrate monotony derives from the usually lower prevalence of IBD in rural or less developed communities consuming locally-produced seasonal products [16], in contrast to the modern Westernized diet, for which the digestive tract would theoretically not have had time to adapt [188]. 

In terms of dietary supplements, vitamin D appears to have a role in IBD. Vitamin D3 (animal sources and skin exposure to ultraviolet light) and vitamin D2 (plant sources) are first hydroxylated in the liver, and then in the kidney and extrarenal tissues, including intestinal macrophages, into the active form 1,25-dihydroxyvitamin D. The active vitamin D binds to its specific receptor in different tissues, including immune cells, modulating gene expression. Vitamin D deficiency has been frequently seen in patients with IBD; therefore, it has been regarded as a protective factor against disease development [189,190]. Particularly in patients with CD, vitamin D deficiency has been consistently associated with disease activity [79,191,192]. Vitamin D3 supplementation for patients with CD has been suggested to alleviate clinical manifestations [193]. In another clinical investigation, a randomized double-blind placebo-controlled study, dietary supplementation with 1200 IU vitamin D3 daily for 12 months significantly increased the vitamin D levels, but only modestly reduced the risk of disease relapse [194]. 

Currently, the exact mechanisms by which vitamin D may reduce the severity of CD are still unclear. In a study using peripheral blood mononuclear cells, investigators found that vitamin D was able to reduce the expression of pro-inflammatory M1 cytokines but did not induce the anti-inflammatory M2 phenotype [195]. In other experimental studies, vitamin D deficiency, or even its impaired signaling, were shown to worsen different models of experimental colitis through multiple effects, including epithelial barrier disruption [196,197], reduced mucosal immunity [198], and an association with gut dysbiosis [199,200].

## 10. Future Directions

Among the environmental changes associated with the progressive and global expansion of IBD, diet has emerged as an essential regulatory element of the gut microbiome, which in turn has been strongly associated with the pathogenesis of several complex chronic inflammatory and autoimmune disorders. In addition to the involvement in dysbiosis, dietary components have been shown to interfere in intestinal homeostasis, modulating the barrier function and innate immunity mechanisms. Therefore, the accumulated scientific evidence appears to position diet as a central element both in disease pathogenesis as a critical risk factor and also as a potential opportunity for therapeutic interventions. Currently, several clinical trials are being conducted in different institutions and, hopefully, novel approaches to dietary therapy will be available in the near future. 

Considering the progressive burden due to IBD anticipated in the next decades, dietary interventions appear particularly interesting, presumably due to the expected irrelevant side effects, lower costs, and potential application in the prevention of disease initiation or relapses. For the moment, however, the simplest approach includes the avoidance of foods that patients self-identify as worsening their symptoms, in addition to high-fat, high-carbohydrate meals and processed foods. Notably, dietary measures should be considered within the wider context of the patient’s routine, not only in terms of food quality, but also changes in lifestyle, including dietary habits. For this purpose, while the results of large prospective controlled studies are awaited to provide more specific dietary guidance to patients, it is important to reinforce the role of multidisciplinary teams, including nutritionists, for offering better attention to and individualized support for patients with IBD.

## Figures and Tables

**Table 1:** Relationship between gut microbiota alterations and dietary patterns.

Genus	Characteristics	Dietary Component	Gastrointestinal (GI) Effect
*Prevotella* *Bacteroides*	Gram-negative anaerobic	Diets rich in fat and carbohydrates	Involved in colitis [17,20,21,22,23]
*Clostridium*	Gram-positive anaerobic	Decrease in high-fat but not high-sugar diets	Affects the clinical course of inflammatory bowel disease (IBD) [21,22,23,24]
*Ruminococcus*	Gram-positive anaerobic	Fermentation of dietary fiber and decrease in high-fat but not high-sugar diets	[21,25,26]
*Streptococcus Lactobacillus*	Gram-positive	Constituents of the normal animal microbiota	Induces remission in ulcerative colitis (UC) patients [21]
*Escherichia*	Gram-negative anaerobic	High-fat and high-sugar diets	Overgrowth of *Escherichia coli* and involved in colitis [16,22,27]
Bifidobacterium	Gram-positive anaerobic	High-fiber diet	Induces remission in UC patients [28,29]
Fusobacterium	Gram-negative anaerobic	-	Involved in colitis and colon cancer [30]
-	-	Alcohol	Decreased butyrate/total short chain fatty acids (SCFA) ratio in stool [31]

Source: adapted from Levine et al. [5] and Rapozo et al. [21].

**Table 2:** Dietary patterns potentially affecting microbiota and immunity.

Dietary Component	GI and Immunological Effect
High-fat, high-sugar diet	Intestinal mucosa dysbiosis [41] Higher degree of crypt abscesses Decreased butyrate production Overgrowth of *E. coli* [41]
Milk fat (saturated fat)	Induced pro-inflammatory Th1 immune response [61] Increased incidence of colitis [61] Dysbiosis
High-fiber diet	Protected from acute colitis [62] Did not increase SCFAs production in patients with UC [62]
Animal versus plant diet	Animal-based food increased the abundance of bile-tolerant microorganisms and decreased the proportions of *Firmicutes* [27]
Alcohol	Decreased butyrate/total SCFA ratio in stool [31]
Maltodextrin	Biofilm formation of adherent invasive *E. coli* [63,64] Enhanced *E. coli* adhesion independent of cellular receptor CEACAM 6 [63,64]

Source: adapted from Levine et al. [5].
